# Regional differences of macrovascular disease in Northeast and South Germany: the population-based SHIP-TREND and KORA-F4 studies

**DOI:** 10.1186/s12889-018-6265-0

**Published:** 2018-12-03

**Authors:** Violetta Ptushkina, Esther Jacobs, Sabine Schipf, Henry Völzke, Marcello Ricardo Paulista Markus, Matthias Nauck, Christa Meisinger, Annette Peters, Werner Maier, Christian Herder, Michael Roden, Wolfgang Rathmann

**Affiliations:** 1 Institute for Biometrics and Epidemiology, German Diabetes Center (DDZ), Leibniz Center for Diabetes Research at Heinrich Heine University Düsseldorf, Auf’m Hennekamp 65, 40225 Düsseldorf, Germany; 2 Institute for Community Medicine, University Medicine Greifswald, Greifswald, Germany; 3 German Center for Cardiovascular Research (DZHK), Partner Site Greifswald, Greifswald, Germany; 4 Department of Internal Medicine B, University Medicine Greifswald, Greifswald, Germany; 5 Institute of Clinical Chemistry and Laboratory Medicine, University Medicine Greifswald, Greifswald, Germany; 6 Institute of Epidemiology II, Helmholtz Zentrum München, German Research Center for Environmental Health (GmbH), Neuherberg, Germany; 7 Institute of Health Economics and Health Care Management, Helmholtz Zentrum München, German Research Center for Environmental Health (GmbH), Neuherberg, Germany; 8 Division of Endocrinology and Diabetology, Medical Faculty, Heinrich Heine University Düsseldorf, Düsseldorf, Germany; 9 Institute for Clinical Diabetology, German Diabetes Center (DDZ), Leibniz Center for Diabetes Research at Heinrich Heine University Düsseldorf, Düsseldorf, Germany; 10 German Center for Diabetes Research (DZD e.V.), Neuherberg, Germany; 11 Medical Faculty, Heinrich Heine University Düsseldorf, Düsseldorf, Germany

**Keywords:** Type 2 diabetes, Macrovascular disease, Regional differences, Prevalence, Population-based studies

## Abstract

**Background:**

Previous studies found regional differences in the prevalence and incidence of type 2 diabetes between Northeast and South of Germany. The aim of this study was to investigate if regional variations are also present for macrovascular disease in people with type 2 diabetes and in the general population. A further aim was to investigate if traditional risk factors of macrovascular complications can explain these regional variations.

**Methods:**

Data of persons aged 30–79 from two regional population-based studies, SHIP-TREND (Northeast Germany, 2008–2012, *n* = 2539) and KORA-F4 (South Germany, 2006–2008, *n* = 2932), were analysed. Macrovascular disease was defined by self-reported previous myocardial infarction, stroke or coronary angiography. Multivariable logistic regression was performed to estimate odds ratios (OR) and 95% confidence intervals (CI) for prevalence of macrovascular disease in persons with type 2 diabetes and in the general population.

**Results:**

The prevalence of macrovascular disease in persons with type 2 diabetes and in the general population was considerably higher in the Northeast (SHIP-TREND: 32.8 and 12.0%) than in the South of Germany (KORA-F4: 24.9 and 8.8%), respectively. The odds of macrovascular disease in persons with type 2 diabetes was 1.66 (95% CI: 1.11–2.49) in the Northeast in comparison to the South after adjustment for sex, age, body mass index, hypertension, hyperlipidemia and smoking. In the general population, SHIP-TREND participants also had a significantly increased odds of macrovascular disease compared to KORA-F4 participants (OR = 1.63, 95% CI: 1.33–2.00). After excluding coronary angiography (myocardial infarction or stroke only), the ORs for region decreased in all models, but the difference between SHIP-TREND and KORA-F4 participants was still significant in the age- and sex-adjusted model for the general population (OR = 1.34, 95% CI: 1.01–1.78).

**Conclusions:**

This study provides an indication for regional differences in macrovascular disease, which is not explained by traditional risk factors. Further examinations of other risk factors, such as regional deprivation or geographical variations in medical care services are needed.

## Background

The prevalence of type 2 diabetes rises steadily all over the world [1, 2]. In 2014, there were considerable regional differences in the prevalence of type 2 diabetes in Europe ranging from 2.4% in Moldova to 14.9% in Turkey [3]. Interestingly, the prevalence of type 2 diabetes shows regional variations even within Germany [4]. In a meta-analysis of regional population-based surveys, a substantially higher prevalence of 12.0% in the East (CARLA) and 10.9% in the Northeast (SHIP) was reported, compared to 5.8% in the South (KORA-S4) of Germany [4]. Incidence of type 2 diabetes exhibits similar differences being highest in the East and lowest in the South of Germany [5]. Nevertheless, it remains unclear, whether more frequently performed screening tests or a higher morbidity led to a higher diabetes prevalence and incidence in the Northeast than in other regions. However, according to pooled population-based data from the same regions of Germany, there is also a considerably higher prevalence of prediabetes and undiagnosed type 2 diabetes in the Northeast than in the South [6].

Patients with type 2 diabetes are at increased risk for diabetes-related macrovascular complications, such as coronary heart disease, stroke or peripheral vascular disease [7, 8]. These complications lead to a reduced quality of life and premature death [9, 10]. Altogether, diabetes-related complications represent a growing social and economic health problem [11]. Knowledge about regional differences in macrovascular disease and related risk factors are necessary to identify high risk groups and to plan specific strategies for its prevention.

The aim of this analysis was to investigate, if regional variations are also existent for the prevalence of macrovascular disease in people with type 2 diabetes and in the general population. A further aim was to investigate if traditional risk factors of macrovascular complications can explain these regional differences.

## Methods

### Study populations

#### The SHIP-TREND study (Northeast of Germany)

The SHIP-TREND study region is located in the Northeast of Germany, the part of the former German Democratic Republic (GDR). A stratified random sample of 8826 adults aged 20–79 years with German nationality was drawn from a central population registry of Western Pomerania (212,157 inhabitants) with the aim to assess prevalence and incidence of common risk factors, subclinical disorders and clinical diseases in the German population. A two-stage cluster sampling method was used which followed the WHO Multinational Monitoring of Trends and Determinants in Cardiovascular Disease (MONICA) Project in Germany. Stratification variables were age, sex, and city/county of residence. Of all persons invited, 4420 (50.1%) individuals participated in the examinations between 2008 and 2012. All participants provided written informed consent and the medical ethics committee of the University of Greifswald approved the study protocol. Further information on the study design of the SHIP-TREND study has been previously published [12].

#### The KORA-F4 study (South of Germany)

The KORA-F4 study (2006–2008) is the 7-year follow-up of the KORA-S4 study (1999–2001), a population-based health survey which was carried out in the city of Augsburg and 16 municipalities from the surrounding counties (about 600,000 inhabitants), which is part of the former Federal Republic of Germany (FRG). The survey sampling method of the former WHO MONICA project was used. Within each selected community, a stratified sample with ten equal strata by sex and age was drawn. As in SHIP-TREND, only individuals with German citizenship and main residency in the study area were included. Of the 4261 participants aged 25–74 years in S4, 3080 took part in the F4 study (72.3%). The loss of participants from S4 to F4 occurred due to deaths (*n* = 176), demands for the deletion of data (*n* = 12), or because participants were completely lost to the follow-up (206), could not be contacted (*n* = 174), were unable to come (*n* = 218) or refused to participate (*n* = 395) [13]. All study participants gave written informed consent to the study. The study design was approved by the ethics committee of the Bavarian Medical Association. The study design, sampling method and data collection have been described in detail elsewhere [14].

### Variables

In both studies, information on sociodemographic variables, lifestyle habits and medical history were collected by trained and certified staff during standardized personal interviews [12, 14].

#### Macrovascular disease

Macrovascular disease was defined as having a self-reported previous myocardial infarction, stroke or a coronary angiography and was not validated by a physician’s assessment.

The variable “coronary angiography” did not include participants who also had a previous myocardial infarction. As myocardial infarction is one of the most common indication for a coronary angiography [15], the participants who had a myocardial infarction were most probably already included in the corresponding variable.

#### Diabetes and glucose tolerance

Known type 2 diabetes was defined using self-reported physician’s diagnosis of diabetes. In KORA-F4, this information was validated by a physician [6]. The 75-g oral glucose tolerance tests (OGTT) were carried out in all participants without known diabetes according to concordant standardised operating procedures in both studies [14]. However, measurements of fasting glucose and 2-h glucose were based on plasma in SHIP-TREND and on serum in KORA-F4. To examine whether the values are comparable, duplicate measurements were performed using serum samples of all SHIP-TREND participants. The analysis revealed, that both measurements were highly correlated (*r* = 0.99, *p* < 0.0001) and showed a very good concordance [6]. Moreover, 30 serum blood glucose samples were randomly taken from KORA-F4 and reassessed in the SHIP-TREND laboratory; the calculated correlation coefficient was 0.94 (*p* < 0.0001). On average, these original 30 KORA-F4 measurements were only slightly lower (mean − 0.06 mmol/l; SD = 0.17) [6]. Hence, it was decided to regard the serum glucose values from KORA-F4 and plasma glucose values from SHIP-TREND as comparable for the current study.

Depending on the OGTT results, participants were categorised to three distinctive groups [16]: Normal glucose tolerance (fasting glucose values < 5.6 mmol/l (< 100 mg/dl) and 2-h glucose < 7.8 mmol/l (< 140 mg/dl)), prediabetes (fasting glucose 5.6–6.9 mmol/l (100–125 mg/dl) and/or 2-h glucose values 7.8–11.0 mmol/l (140–199 mg/dl)) and newly diagnosed diabetes (fasting glucose values ≥7.0 mmol/l (≥ 126 mg/dl) or 2-h glucose ≥11.1 mmol/l (≥ 200 mg/dl)).

#### Medical data

Anthropometric measurements were taken after removing shoes, heavy clothing and belts. Body mass index (BMI) was calculated as weight [kg] divided by height^2^ [m^2^]. Blood pressure measurements were taken at the right arm after a rest period of at least five minutes in a sitting position and repeated three times at an interval of three minutes. The mean of the second and third measurement was calculated. A fasting venous blood sample was obtained from all study participants while sitting. Triglycerides were measured with the GPO-PAP-method (TGL Flex, Dade Behring, Marburg, Germany). Plasma high-sensitivity C-reactive protein (hsCRP) concentrations were measured using a latex enhanced nephelometric assay run on a BN II analyser (Dade Behring, Marburg, Germany). HsCRP values higher than 10 mg/l were excluded from the analysis to include only participants with subclinical inflammation. Glycated hemoglobin (HbA1c) was measured by high-performance liquid chromatography with spectrophotometric detection (Diamat Analyzer; Bio-Rad, Munich, Germany) and a coefficient of variation of 1.5%. The insulin resistance score (HOMA-IR) was calculated as fasting plasma glucose (mmol/l) × fasting serum insulin (mlU/l) / 22.5.

#### Hypertension and hyperlipidemia

During the interview, the participants were asked whether a doctor had ever diagnosed increased/high blood pressure or increased blood lipids in the last 12 months. Furthermore, they were asked to bring original packaging of their medications used during the last 7 days. Finally, the variables “hypertension” and “hyperlipidemia” were defined as follows: hypertension as self-reported increased/high blood pressure or intake of antihypertensive drugs, hyperlipidemia as self-reported increased blood lipids or intake of lipid-lowering drugs.

#### Educational level

In both studies, the participants were asked for their highest school degree achieved. Educational level was defined as low and middle (less than university qualification) or high level (university qualification). According to the German school system, low educational level includes participants with up to 9 years of schooling. Middle educational level is equivalent to 10 years of schooling and high educational level to 12 or 13 years of schooling, which is the general qualification for university entrance [17].

#### Physical activity

Physical activity during leisure time was assessed by self-report. Participants were classified as active if they reported regular participation in sports in summer and winter and being active for > 1 h per week in both seasons [18].

#### Smoking

Three categories (current, ex- and non-smoker) were defined. Participants were classified as current smokers if they smoked at least one cigarette per day regularly, or if they had stopped smoking less than 12 months ago. Persons were defined as ex-smokers if they had stopped smoking more than 12 months ago and as non-smokers if they had either never smoked or less than one cigarette per day [18, 19].

### Study sample

The converged data set contained participants aged 30 to 79 years (SHIP-TREND: *n* = 3960, KORA-F4: *n* = 3022). Participants were excluded from the analysis if they had type 1 diabetes (SHIP-TREND: *n* = 62, KORA-F4: *n* = 9) and missing information on known diabetes or missing or implausible values in glucose measurements (SHIP-TREND: *n* = 615, KORA-F4: *n* = 81). All participants who underwent glucose tolerance testing were instructed to fast for at least 8 h prior to the test. In SHIP-TREND, however, 744 participants without known diabetes did not fulfill this requirement. These participants were also excluded from the analysis. Therefore, the present study is based on 2539 SHIP-TREND (Northeast) and 2932 KORA-F4 (South) participants.

### Statistical analyses

For descriptive statistics, means and standard deviations (SD) were calculated for normally distributed continuous variables, while variables with skewed distribution were described as medians and interquartile ranges. For categorical variables percentages are reported. Because triglycerides, hsCRP and HOMA-IR were not normally distributed, these variables were transformed using the log function to correct the skewness. Differences between groups were assessed using *T*-test for continuous variables and Chi-square test or univariate logistic regression model for categorical variables. The *p*-values for study differences in components of metabolic syndrome were corrected using the step-down Bonferroni procedure to account for multiple comparisons (level of significance *p* < 0.05). Logistic regression models were used to identify factors related to macrovascular disease. Three different models were fitted: model A adjusted for sex and age, model B adjusted for sex, age, BMI, hsCRP, hypertension, hyperlipidemia, smoking, education and physical activity, and model C adjusted for significant variables in models B and the OGTT groups. All models were separately fitted for participants with known type 2 diabetes and for the general population, respectively. Sensitivity analyses (using BMI as a continuous instead of a categorical variable and definition of “macrovascular disease” as having myocardial infarction or stroke only) were performed to test the robustness of conclusions. A two-sided alpha level of 0.05 was chosen as criterion for statistical significance. All analyses were carried out using SAS, version 9.4 (SAS Institute Inc., Cary, NC, USA).

## Results

### Participants’ characteristics stratified by cohort and diabetes status

The baseline characteristics of the participants stratified by cohort are presented in Table 1. SHIP-TREND participants (Northeast) were somewhat younger than KORA-F4 participants (South) and were diagnosed with having type 2 diabetes at an earlier age, whereas the sex distribution was similar. Furthermore, SHIP-TREND participants had a higher BMI, blood pressure values, triglycerides and hsCRP, as well as higher fasting and 2-h glucose values. Participants of both regions slightly differed in mean HbA1c, total and LDL-cholesterol, but not in HDL-cholesterol. Moreover, SHIP-TREND participants reported current smoking significantly more often than KORA-F4 participants. Low and middle educational levels were more frequently found in KORA-F4. The prevalence of macrovascular disease among participants with known diabetes and in the general population was significantly higher in SHIP-TREND than in KORA-F4.

**Table 1:** Baseline participant characteristics by cohort and *p*-values for study differences

	SHIP-TREND	KORA-F4	*p*-value
N	2539	2932	
Age (years)	53.9 (12.9)	55.7 (12.8)	**< 0.001**
Female sex (%)	52.8	51.6	0.373
Middle/low education (%)	73.5	76.3	**0.021**
Age at diagnosis of diabetes (years)^a^	55.2 (10.5)	59.1 (10.1)	**< 0.001**
Body mass index (kg/m^2^)	28.6 (5.3)	27.6 (4.8)	**< 0.001**
No physical activity (%)	48.6	45.0	**0.009**
Current smoking (%)	19.6	16.6	0.064
Ex-smoking (%)	21.4	26.5	**0.001**
Systolic blood pressure (mmHg)	127.7 (18.2)	122.2 (18.6)	**< 0.001**
Diastolic blood pressure (mmHg)	77.3 (10.0)	75.2 (10.0)	**< 0.001**
Hypertension in general population (%)	52.9	50.8	0.125
Self-reported high blood pressure (ever) (%)	48.9	48.6	0.823
Antihypertensive medication (%)	38.4	29.8	**< 0.001**
Hypertension in known diabetes (%)^a^	88.9	88.2	0.782
Self-reported high blood pressure (ever) (%)	80.0	84.8	0.141
Antihypertensive medication (%)	79.2	75.1	0.237
Hyperlipidemia in general population (%)	27.6	30.5	**0.020**
Self-reported high lipid levels (last 12 months) (%)	24.5	28.2	**0.002**
Lipid-lowering medication (%)	14.2	12.3	**0.042**
Hyperlipidemia in known diabetes (%)^a^	59.6	59.5	0.988
Self-reported high lipid levels (last 12 months) (%)	46.0	48.6	0.539
Lipid-lowering medication (%)	41.8	39.2	0.529
Macrovascular disease in general population (%)	12.0	8.8	**< 0.001**
Myocardial infarction (%)	2.9	2.9	0.976
Stroke (%)	1.5	1.0	0.052
Coronary angiography (%)^b^	8.6	5.2	**< 0.001**
Macrovascular disease in known diabetes (%)^a^	32.8	24.9	**0.040**
Myocardial infarction (%)	9.4	11.0	0.544
Stroke (%)	4.7	2.5	0.172
Coronary angiography (%)^b^	22.2	12.3	**0.002**
Fasting glucose (mmol/l)^c^	5.5 (0.8)	5.3 (0.6)	**< 0.001**
2-h glucose (mmol/l)^c^	6.6 (2.4)	6.2 (2.0)	**< 0.001**
HbA1c (%)	5.46 (0.89)	5.55 (0.61)	**< 0.001**
HbA1c (mmol/mol)	36.1 (9.7)	37.1 (6.7)	**< 0.001**
HOMA-IR^d^	2.5 (1.6–3.8)	2.0 (1.4–3.0)	**< 0.001**
Total cholesterol (mmol/l)	5.5 (1.1)	5.6 (1.0)	**0.009**
HDL-cholesterol (mmol/l)	1.4 (0.4)	1.4 (0.4)	0.167
LDL-cholesterol (mmol/l)	3.4 (0.9)	3.5 (0.9)	**< 0.001**
Triglycerides (mmol/l)	1.3 (0.9–1.9)	1.2 (0.8–1.7)	**< 0.001**
hsCRP (mg/l)^e^	1.2 (0.7–2.6)	1.1 (0.6–2.4)	**< 0.001**
eGFR (ml/min/1.73 m^2^)	86.9 (20.1)	88.3 (16.5)	**0.005**

Results are means (± SD), percentages or medians (IQR)

^a^SHIP-TREND: *N* = 344, KORA-F4: *N* = 237

^b^Participants with previous myocardial infarction were excluded

^c^Participants without known diabetes, SHIP-TREND: *N* = 2178, KORA-F4: *N* = 2695

^d^Participants without known diabetes, SHIP-TREND: *N* = 1953, KORA-F4: *N* = 2665

^e^high-sensitivity C - reactive protein. Participants with hsCRP > 10 mg/l were excluded, SHIP-TREND: *N* = 175, KORA-F4: *N* = 112

Significant regional differences (*p* < 0.05) are highlighted in bold

Risk factors, which are associated with cardiovascular disease (metabolic syndrome) [20], stratified by OGTT-status (normal glucose tolerance, prediabetes, newly diagnosed diabetes and known diabetes) are reported in Table 2. The prevalence of prediabetes, newly diagnosed diabetes and known diabetes was higher in SHIP-TREND participants (Northeast) than in KORA-F4 participants (South). The proportions of participants with macrovascular disease were also higher in the Northeast compared to the South in people with normal glucose tolerance, newly diagnosed diabetes and known diabetes, but not in persons with prediabetes. The results of Table 2 confirmed the expectation that the prevalence of risk factors associated with cardiovascular disease increase with increasing blood glucose levels.

**Table 2:** Components of metabolic syndrome by cohort, categorised by normal glucose tolerance, prediabetes, newly diagnosed diabetes and known diabetes

	Normal glucose tolerance	Prediabetes	Newly diagnosed diabetes	Known diabetes
Total % (n)				
KORA-F4	60.5 (1774)	28.4 (834)	3.0 (87)	8.1 (237)
SHIP-TREND	45.3 (1151)	34.9 (885)	5.6 (142)	14.2 (361)
Age (years) mean (std)				
KORA-F4	**51.5 (12.2)**	**60.6 (11.3)**	64.8 (9.7)	**66.9 (8.6)**
SHIP-TREND	**48.6 (12.3)**	**55.2 (11.7)**	61.6 (9.3)	**65.0 (9.3)**
Body mass index (kg/m^2^) mean (SD)				
KORA-F4	26.3 (4.3)	29.1 (4.7)	31.1 (4.4)	**31.5 (5.4)**
SHIP-TREND	26.3 (4.3)	29.3 (4.7)	31.3 (5.2)	**33.0 (5.8)**
Systolic blood pressure (mmHg) mean (SD)				
KORA-F4	**117.4 (16.9)**	**128.1 (18.5)**	132.6 (16.8)	**132.7 (19.6)**
SHIP-TREND	**121.0 (15.7)**	**130.7 (16.8)**	135.7 (19.3)	**138.5 (19.9)**
Diastolic blood pressure (mmHg) mean (SD)				
KORA-F4	**74.2 (9.6)**	**77.3 (10.2)**	77.9 (10.5)	**74.6 (10.5)**
SHIP-TREND	**75.3 (9.4)**	**79.3 (9.7)**	79.1 (10.8)	**78.4 (10.8)**
Fasting glucose (mmol/l)				
KORA-F4	**5.0 (0.3)**	**5.7 (0.5)**	**6.7 (1.3)**	NA
SHIP-TREND	**5.1 (0.3)**	**5.9 (0.4)**	**7.3 (1.8)**	NA
2-h glucose (mmol/l)				
KORA-F4	**5.3 (1.1)**	**7.3 (1.8)**	12.0 (2.8)	NA
SHIP-TREND	**5.5 (1.1)**	**7.1 (1.7)**	12.6 (3.5)	NA
HbA1c (%) mean (SD)				
KORA-F4	**5.3 (0.3)**	**5.6 (0.3)**	6.1 (0.7)	6.8 (1.1)
SHIP-TREND	**5.1 (0.5)**	**5.3 (0.5)**	5.9 (1.0)	6.7 (1.3)
HbA1c (mmol/mol) mean (SD)				
KORA-F4	**34.7 (3.2)**	**37.6 (3.7)**	43.2 (7.6)	51.1 (12.5)
SHIP-TREND	**32.3 (5.9)**	**34.7 (5.6)**	41.1 (10.8)	49.8 (13.7)
HOMA-IR median (IQR)				
KORA-F4	**1.7 (1.2–2.3)**	**2.8 (2.0–4.2)**	5.3 (3.2–7.3)	5.2 (3.5–8.1)
SHIP-TREND	**1.9 (1.3–2.6)**	**3.2 (2.3–4.6)**	5.9 (3.7–8.4)	NA
HDL-cholesterol (mmol/l) mean (SD)				
KORA-F4	1.5 (0.4)	1.4 (0.4)	1.3 (0.3)	1.2 (0.3)
SHIP-TREND	1.5 (0.4)	1.4 (0.3)	1.3 (0.4)	1.2 (0.3)
Triglycerides (mmol/l) median (IQR)				
KORA-F4	**1.0 (0.7–1.5)**	1.4 (1.0–2.0)	1.8 (1.2–2.6)	**1.6 (1.1–2.3)**
SHIP-TREND	**1.1 (0.8–1.5)**	1.4 (1.1–2.0)	1.9 (1.3–2.8)	**2.1 (1.5–3.0)**
hsCRP (mg/l)^a^ median (IQR)				
KORA-F4	**0.9 (0.5–1.9)**	1.4 (0.7–2.7)	2.8 (1.2–4.7)	1.8 (0.9–3.2)
SHIP-TREND	**1.0 (0.6–2.1)**	1.3 (0.7–2.5)	1.9 (0.9–3.7)	2.1 (1.0–4.2)
Macrovascular disease % (n)				
KORA-F4	5.3 (94)	11.1 (92)	12.6 (11)	24.9 (59)
SHIP-TREND	6.1 (70)	10.0 (88)	19.7 (28)	32.8 (118)

Results are means (± SD), percentages or medians (IQR)

*NA* not assessed

^a^High-sensitivity C - reactive protein. Participants with hsCRP > 10 mg/l were excluded, SHIP-TREND: *N* = 175, KORA-F4: *N* = 112

Significant *p*-values for study differences using the step-down Bonferroni correction (the level of significance *p* < 0.05) are highlighted in bold

Mean total cholesterol was similar in both studies in the normal glucose tolerance group (5.5 mmol/l), in people with prediabetes (5.7 mmol/l) and in the newly diagnosed diabetes group (5.6 mmol/l), respectively. In participants with known diabetes, it was slightly lower in SHIP-TREND (5.1 mmol/l) than in KORA-F4 (5.3 mmol/l). LDL-cholesterol was also somewhat lower in SHIP-TREND than in KORA-F4 in all OGTT groups. Insufficient physical activity (less than 1 h/week) was more common in participants in the Northeast (SHIP-TREND), except in the group of known diabetes (54% in SHIP-TREND vs. 62% in KORA-F4). The prevalence of current smokers was higher in SHIP-TREND, but not in the group of newly diagnosed diabetes (12% in SHIP-TREND, 14% in KORA-F4). The percentages of participants who smoked were highest in the group of normal glucose tolerance (24% in SHIP-TREND, 20% in KORA-F4) and lowest in the group of newly diagnosed diabetes in SHIP-TREND (12%) and in the group of known diabetes in KORA-F4 (11%). The prevalence of people who had stopped smoking was highest in groups of newly diagnosed diabetes (20% in SHIP-TREND, 36% in KORA-F4) and known diabetes (32% in SHIP-TREND, 35% in KORA-F4), and lowest in people with normal glucose tolerance (17% in SHIP-TREND, 22% in KORA-F4). Low or middle educational status tended to be more often in participants with known diabetes (about 86% in both studies) and less frequently in people with normal glucose tolerance (70% in SHIP-TREND and 72% in KORA-F4).

### Regional differences and risk factors of macrovascular disease

We performed logistic regression analyses with macrovascular disease as dependent variable separately in persons with known diabetes and in the general population (Table 3). In a model adjusted for sex and age (model A), SHIP-TREND participants (Northeast) with known diabetes had a higher odds (odds ratio (OR)) (OR = 1.66; 95% CI 1.14–2.43) of having macrovascular disease compared to KORA-F4 participants (South). When the model was further adjusted for BMI, hsCRP, hypertension, hyperlipidemia, smoking, education and physical activity (model B), the OR slightly increased to 1.70 (95% CI 1.11–2.62). In the final model, adjusted for sex, age, BMI, hypertension, hyperlipidemia and smoking (model C), SHIP-TREND participants still had significantly increased odds of having macrovascular disease compared to KORA-F4 participants (OR = 1.66, 95% CI 1.11–2.49). In this final model, age, hypertension and hyperlipidemia were significantly positively related to macrovascular disease.

**Table 3:** Multivariate logistic regression analysis for association between macrovascular disease, geographical region and risk factors in people with known diabetes and in the general population

Variable	Macrovascular disease (yes vs. no) Known diabetes		Macrovascular disease (yes vs. no) General population	
	OR (95% Cl)	*p*-value	OR (95% Cl)	*p*-value
Model A				
SHIP-TREND (vs. KORA-F4)	**1.66 (1.14–2.43)**	**0.009**	**1.76 (1.46–2.12)**	**< 0.001**
Men (vs. women)	**1.73 (1.18–2.52)**	**0.005**	**2.20 (1.81–2.67)**	**< 0.001**
Age (per year, continuous)	**1.05 (1.03–1.07)**	**< 0.001**	**1.09 (1.08–1.10)**	**< 0.001**
Model B				
SHIP-TREND (vs. KORA-F4)	**1.70 (1.11–2.62)**	**0.015**	**1.78 (1.44–2.19)**	**< 0.001**
Men (vs. women)	1.50 (0.93–2.41)	0.098	**1.97 (1.57–2.47)**	**< 0.001**
Age (per year, continuous)	**1.05 (1.02–1.08)**	**0.001**	**1.07 (1.05–1.08)**	**< 0.001**
BMI ≥ 25 kg/m^2^ (vs. < 25 kg/m^2^)	2.55 (0.85–7.65)	0.096	**1.39 (1.01–1.93)**	**0.046**
BMI ≥ 30 kg/m^2^ (vs. < 25 kg/m2)	1.91 (0.65–5.64)	0.241	1.14 (0.80–1.61)	0.466
hsCRP (mg/l)^a^ (continuous)	1.15 (0.90–1.47)	0.254	1.04 (0.92–1.17)	0.534
Hypertension (vs. no)	2.35 (0.94–5.88)	0.069	**3.47 (2.57–4.68)**	**< 0.001**
Hyperlipidemia (vs. no)	**3.86 (2.41–6.18)**	**< 0.001**	**3.34 (2.70–4.14)**	**< 0.001**
Smoker (vs. never smoker)	1.47 (0.73–2.97)	0.278	1.16 (0.81–1.64)	0.417
Ex-smoker (vs. never-smoker)	1.58 (0.98–2.55)	0.061	**1.45 (1.15–1.84)**	**0.002**
Low/middle education (vs. high education)	0.92 (0.51–1.64)	0.767	1.22 (0.94–1.59)	0.134
< 1 h physical activity (vs. > 1 h)	0.96 (0.62–1.47)	0.837	1.01 (0.82–1.24)	0.945
Model C				
SHIP-TREND (vs. KORA-F4)	**1.66 (1.11–2.49)**	**0.014**	**1.63 (1.33–2.00)**	**< 0.001**
Men (vs. women)	1.49 (0.97–2.29)	0.070	**1.89 (1.52–2.34)**	**< 0.001**
Age (per year, continuous)	**1.05 (1.02–1.08)**	**0.001**	**1.06 (1.05–1.08)**	**< 0.001**
BMI ≥ 25 kg/m^2^ (vs. < 25 kg/m^2^)	1.85 (0.67–5.10)	0.237	**1.39 (1.01–1.92)**	**0.041**
BMI ≥ 30 kg/m^2^ (vs. < 25 kg/m2)	1.72 (0.64–4.66)	0.287	1.15 (0.82–1.61)	0.419
Hypertension (vs. no)	**2.79 (1.13–6.87)**	**0.026**	**3.48 (2.59–4.68)**	**< 0.001**
Hyperlipidemia (vs. no)	**3.48 (2.25–5.36)**	**< 0.001**	**3.16 (2.57–3.88)**	**< 0.001**
Smoker (vs. never smoker)	1.27 (0.66–2.45)	0.477	1.10 (0.79–1.54)	0.572
Ex-smoker (vs. never-smoker)	1.51 (0.97–2.34)	0.066	**1.48 (1.18–1.86)**	**0.001**
Known diabetes (vs. normal glucose tolerance)	–	–	**1.37 (1.03–1.84)**	**0.033**
Prediabetes (vs. normal glucose tolerance)	–	–	0.86 (0.67–1.10)	0.226
Newly diagnosed diabetes (vs. normal glucose tolerance)	–	–	0.99 (0.64–1.52)	0.950

*BMI* body mass index, *OR* odds ratio, *CI* confidence interval

^a^High-sensitivity C - reactive protein. Participants with hsCRP > 10 mg/l were excluded, SHIP-TREND: *N* = 175, KORA-F4: *N* = 112

Significant regional differences (*p* < 0.05) are highlighted in bold

Fitting logistic regression models, adjusting for age and sex and including all study participants (model A), we found that persons in the Northeast (SHIP-TREND) also had a significantly higher odds for having myocardial infarction, stroke or coronary angiography (OR = 1.76, 95% CI 1.46–2.12) compared to participants in the South (KORA-F4). In model B, adjusted for further risk factors, the OR for region increased to 1.78 (95% CI 1.44–2.19). In model C, adjusted for sex, age, BMI, hypertension, hyperlipidemia, smoking and OGTT groups (known diabetes, prediabetes, newly diagnosed diabetes, normal glucose tolerance as reference), the odds slightly decreased to 1.63 (95% CI 1.33–2.00). In the general population, known diabetes was associated with an increased odds of having macrovascular disease (OR = 1.37, 95% CI 1.03–1.84) compared to participants with normal glucose tolerance. Sex, age, BMI, hypertension, hyperlipidemia and smoking were also significantly positively related to macrovascular complications.

### Sensitivity analyses

First, a sensitivity analyses was carried out to evaluate the effect of using BMI as a continuous instead of a categorical variable for obesity. The OR for macrovascular disease in SHIP-TREND participants (Northeast) compared to KORA-F4 participants (South) remained almost unchanged in all models. In the second sensitivity analysis (Table 4), we excluded coronary angiography from the definition of “macrovascular disease” (myocardial infarction or stroke only). The ORs for region decreased in all models, but the difference between SHIP-TREND and KORA-F4 participants was still significant in the age- and sex-adjusted model for the general population (OR = 1.34, 95% CI 1.01–1.78). We did not find any interaction of the variable “OGTT group” (normal glucose tolerance, prediabetes, newly diagnosed diabetes or known diabetes) and cohort (SHIP-TREND, KORA-F4) in people with prediabetes, known diabetes as well as for newly diagnosed diabetes.

**Table 4:** Multivariate logistic regression analysis for association between myocardial infarction or stroke, geographical region and risk factors

Variable	Myocardial infarction or stroke (yes vs. no) Known diabetes^a^		Myocardial infarction or stroke (yes vs. no) General population^b^	
	OR (95% Cl)	*p*-value	OR (95% Cl)	*p*-value
Model A				
SHIP-TREND (vs. KORA-F4)	1.14 (0.69–1.89)	0.600	**1.34 (1.01–1.78)**	**0.004**
Men (vs. women)	**2.05 (1.20–3.51)**	**0.009**	**2.58 (1.90–3.51)**	**< 0.001**
Age (per year, continuous)	**1.07 (1.04–1.11)**	**< 0.001**	**1.10 (1.09–1.12)**	**< 0.001**
Model B				
SHIP-TREND (vs. KORA-F4)	1.09 (0.62–1.94)	0.764	1.31 (0.95–1.79)	0.098
Men (vs. women)	1.92 (0.96–3.84)	0.065	**2.30 (1.60–3.31)**	**< 0.001**
Age (per year, continuous)	**1.10 (1.05–1.14)**	**< 0.001**	**1.08 (1.06–1.10)**	**< 0.001**
BMI ≥ 25 kg/m^2^ (vs. < 25 kg/m^2^)	1.53 (0.38–6.23)	0.555	1.08 (0.66–1.80)	0.753
BMI ≥ 30 kg/m^2^ (vs. < 25 kg/m2)	1.60 (0.40–6.36)	0.506	0.93 (0.55–1.58)	0.782
hsCRP (mg/l)^c^ (continuous)	1.30 (0.93–1.81)	0.121	1.09 (0.91–1.31)	0.344
Hypertension (vs. no)	1.29 (0.35–4.67)	0.703	3.58 (2.08–6.19)	**< 0.001**
Hyperlipidemia (vs. no)	**4.27 (2.08–8.77)**	**< 0.001**	4.76 (3.31–6.85)	**< 0.001**
Smoker (vs. never smoker)	**2.93 (1.18–7.27)**	**0.021**	**1.96 (1.17–3.29)**	**0.012**
Ex-smoker (vs. never-smoker)	1.48 (0.77–2.86)	0.243	**1.69 (1.18–2.42)**	**0.004**
Low/middle education (vs. high education)	1.17 (0.52–2.64)	0.699	**1.68 (1.08–2.61)**	**0.022**
< 1 h physical activity (vs. > 1 h)	0.80 (0.45–1.43)	0.451	1.16 (0.85–1.60)	0.355
Model C				
SHIP-TREND (vs. KORA-F4)	1.11 (0.66–1.87)	0.689	1.21 (0.89–1.64)	0.229
Men (vs. women)	1.74 (0.96–3.16)	0.071	**2.23 (1.58–3.14)**	**< 0.001**
Age (per year, continuous)	**1.08 (1.04–1.12)**	**< 0.001**	**1.08 (1.06–1.09)**	**< 0.001**
Hypertension (vs. no)	2.07 (0.61–7.08)	0.245	**3.32 (1.99–5.56)**	**< 0.001**
Hyperlipidemia (vs. no)	**4.28 (2.21–8.26)**	**< 0.001**	**4.74 (3.35–6.72)**	**< 0.001**
Smoker (vs. never smoker)	1.89 (0.82–4.35)	0.136	**1.72 (1.05–2.81)**	**0.031**
Ex-smoker (vs. never-smoker)	1.35 (0.76–2.42)	0.311	**1.62 (1.16–2.27)**	**0.005**
Low/middle education (vs. high education)	1.43 (0.66–3.13)	0.369	**1.70 (1.12–2.58)**	**0.013**
Known diabetes (vs. normal glucose tolerance)	–	–	1.37 (0.91–2.06)	0.130
Prediabetes (vs. normal glucose tolerance)	–	–	0.93 (0.63–1.36)	0.696
Newly diagnosed diabetes (vs. normal glucose tolerance)	–	–	0.94 (0.49–1.81)	0.862

*BMI* body mass index, *OR* odds ratio, *CI* confidence interval

^a^SHIP-TREND: *N* = 47, KORA-F4: *N* = 31

^b^SHIP-TREND: *N* = 105, KORA-F4: *N* = 109

^c^High-sensitivity C - reactive protein. Participants with hsCRP > 10 mg/l were excluded, SHIP-TREND: *N* = 175, KORA-F4: *N* = 112

Significant regional differences (*p* < 0.05) are highlighted in bold

## Discussion

Our analyses suggested a higher prevalence of macrovascular disease in the Northeast than in the South of Germany in persons with known type 2 diabetes as well as in the general population. SHIP-TREND participants (Northeast) had significantly increased odds of having myocardial infarction, stroke or a coronary angiography compared to KORA-F4 participants (South). After excluding coronary angiography from the outcome definition, significant regional differences for cardiovascular events were still present in the general population. In people with type 2 diabetes, macrovascular disease had a positively significant relationship with higher age, hypertension and hyperlipidemia. In the general population, male sex, higher age, overweight and smoking were positively associated with macrovascular disease. However, these traditional risk factors were not able to fully explain the regional differences.

The regional variation in macrovascular disease within Germany is in line with previous studies on prevalence and mortality of cardiovascular disorders in German federal states [21–24]. The results from the nationwide telephone health survey in Germany (German Health Update (GEDA) from 2009-2012) on regional differences in the prevalence of cardiovascular disease with 62,214 participants showed that the prevalence of myocardial infarction, coronary heart disease, heart failure or stroke varied between nearly 13% in the North and 10% in the South [21]. Furthermore, the data from the Federal Statistical Office of Germany demonstrated age-standardized mortality rates from ischaemic heart disease of 169–177 per 100,000 persons in the Northeast and 95–105 per 100,000 persons in the South [22].

Life-style risk factors are the most important potential explanation for these regional differences. According to the Global health risks report on mortality and burden of disease attributable to major risk factors, physical inactivity, risky alcohol consumption, smoking, low fruit and vegetable consumption, as well as obesity, hypertension and diabetes cause cardiovascular disease in more than half of all cases [25]. North-South differences in these risk factors within Germany has been demonstrated in a number of previous studies [18, 26–31]. The GEDA study reported a prevalence of physical inactivity of 41.4% in men and 41.2% in women in the Northeast and 33.3% in men and 33.1% in women in the South. Furthermore, the prevalence of smoking was 35.1% in men and 23.2% in women in the Northeast compared to 30.6% in men and 23.0% in women in the South. In addition, the prevalence of obesity was 20.0% in men and 21.3% in women in the Northeast and 16.4% in men and 14.7% in women in the South. Finally, the prevalence of hypertension was 38.9% in both sexes in the Northeast and 32.0% in men and 28.0% in women in the South [26]. The data from the population-based German Health Interview and Examination Survey conducted in the years 2008–2011 with 7074 participants reported a prevalence of hypertension of 36.6% in men and 30.5% in women in the Northeast and 31.3% in men and 25.9% in women in the South [30]. Another study published in 2006 on regional differences in smoking among adults in Germany found a prevalence of current smokers of 37.8% in men and 28.5% in women in SHIP (Northeast) and 34.0% in men and 22.4% in women in KORA-S4 (South) [27]. A further national cross-sectional study with 35,869 participants, performed in 2005, showed a higher prevalence of overweight of 39.2% in the Northeast and 36.3% in the South [28]. A further analysis to regional differences in the prevalence of the metabolic syndrome in Germany, based on the same data, showed that the metabolic syndrome was more frequent in the Northeast (26.2%) than in the South of Germany (21.4%) [29]. According to GEDA study, the prevalence of risky alcohol consumption in men was slightly higher in the North than in the South of Germany (34.8% vs. 32.9%) [26]. In our study we were not able to analyse alcohol consumption because of different assessment methods in SHIP-TREND and KORA-F4.

Another aspect that needs to be discussed is that the Northeastern region of SHIP-TREND and the Southern region of KORA-F4 were located in two different states from 1949 to 1990 (GDR and FRG). These states differed considerably in their legislation, economic system, health care [32] as well as cardiovascular risk factors. As an example, people in the GDR had a higher frequency of overweight and obesity, physical inactivity, smoking and consumption of alcohol and soft drinks [24, 33, 34]. The most likely explanation for the higher frequency of overweight and obesity is that the availability of certain foods on the market had been limited in the GDR. As a result, the consumption of vegetable oil, fresh fruits and vegetables was lower and the intake of fatty foods, like sausages much higher [35]. More than 25 years after reunification there are still diversities in risk determinants, which might be of importance for the different prevalence of risk factors for macrovascular disease [24]. Furthermore, the access to healthcare [36] and factors of the physical and built environment [37] may contribute to the differences in mortality and morbidity within Germany. Moreover, the individual socioeconomic status (income, education, occupation) may also play a role, in so far as the occurrence of cardiovascular disease is the highest among individuals in poorer socio-economic circumstances [38]. Further studies showed, that not only individuals with low socio-economic status, but also persons who live in socioeconomically deprived areas are at increased risk of obesity and type 2 diabetes [17, 39]. Finally, mental health could play a crucial role. Various epidemiologic and basic science studies established a connection between cardiovascular disease and depression, psychological stress and anxiety [40]. In the national surveillance by the Robert Koch-Institute (2014) differences in the prevalence of mental disorders between former GDR (Northeast) and former FGR (South) were also reported (36.6% in women and 20.4% in men in former GDR, 33.7% in women and 23.0% in men in former FRG) [24]. Furthermore, routine data from 2000 to 2013 of the Techniker Krankenkasse, one of the biggest statutory health funds in Germany covering about 9% of the population, showed that the number of sick days due to depression was higher in the region of SHIP-TREND than in region of KORA-F4 (109 vs. 90 per 100 employed persons, respectively) [41].

While many studies worldwide reported geographic differences within countries in macrovascular disease or in cardiovascular mortality in the general population [42–49], only few studies reported the regional variations among people with type 2 diabetes. The data from the French national mortality registry from 2002 showed that the burden of diabetes-related mortality was higher in the Northeast than in the West of France [50]. To our knowledge, our research is the first international study, analysing these regional differences in people with type 2 diabetes.

Finally, it is noteworthy that the all-cause mortality as well as the incidence rates for cardiovascular disease among people with type 2 diabetes have fallen considerably in recent years worldwide [51, 52]. Observations from the USA, based on the national surveillance data, showed that the rates of myocardial infarction and stroke in people with type 2 diabetes declined between 1990 and 2010 by 68 and 53%, respectively [53]. Furthermore, a retrospective cohort study from Canada, based on 670,602 people with and 9,190,721 people without diabetes, reported that the rates of patients having acute myocardial infarction or stoke decreased between 1992 and 2000 more in people with diabetes than in people without diabetes [54]. A study from Germany, including 14,578 individuals with myocardial infarction, also showed a significant decrease in the incidence of myocardial infarction in women with diabetes between 1985 and 2006 [55]. These findings suggest that care and management of patients with diabetes has been improved in recent decades, which leads to better primary and secondary prevention of diabetes-related complications [52, 54]. Thus, it was important that the time of data assembling of both surveys (SHIP-TREND, KORA-F4) was comparable.

Interestingly, our analysis revealed that ex-smokers had a greater risk for macrovascular disease than active smokers. A similar observation was reported in a study from Germany on regional variability of lifestyle factors and hypertension with prediabetes and newly diagnosed diabetes [18]. Possible explanations for these observations could be an increased intake of high-caloric food after quitting smoking and the resulting risks, such as overweight [56], or survival bias due to increasing survival of former smokers in comparison to individuals who continue to smoke [57]. Another explanation might be that the ex-smokers had stopped smoking because of a previous heart attack, stroke or coronary heart disease. As both studies are of cross-sectional design, unfortunately cause and effect relationships cannot be detected.

The major strength of our study is a large population-based sample of well-characterised participants including detailed data on lifestyle and multiple risk factors, measured following common standardised protocols in SHIP-TREND and KORA-F4. Several limitations of the study should be mentioned. First, it is possible that the prevalence of macrovascular disease was underestimated in the South of Germany since in the follow-up survey (KORA-F4) persons with higher morbidity most likely had a higher likelihood of not attending the follow-up investigation. Second, in SHIP-TREND the diabetes status was obtained by self-reported physician’s diagnosis only. However, results of several studies showed that the accuracy of self-reports for diabetes is generally high [58, 59]. Furthermore, the measurements of glucose values were based on plasma in SHIP-TREND and serum in KORA-F4. Nevertheless, the measurements were compared in SHIP-TREND and showed a high degree of agreement between both methods. Finally, the SHIP-TREND and KORA-F4 are restricted to participants of German nationality only, so that the results cannot be referred to the current resident population of Germany without further assumptions.

In our analysis we found relatively high prevalence of self-reported high blood pressure (48.9% in SHIP-TREND, 48.6% in KORA-F4), especially in people with diabetes (80.0% in SHIP-TREND, 84.8% in KORA-F4). The most likely explanation is that the blood pressure status was obtained by the question: “Have you *ever* had a diagnosis of an increased or high blood pressure?”, which could have led to an overestimation of the prevalence of current high blood pressure.

Finally, we defined our outcome variable “macrovascular disease” as having previous myocardial infarction, stroke or coronary angiography. Coronary angiography was included in the outcome variable because it is an important indicator for coronary heart disease [15]. The most common indication for a coronary angiography in Germany is angina pectoris (24.3%), followed by acute myocardial infarction (20.1%) and chronic ischemic heart disease (19.6%). Further reasons for catheter examination are sore throat and chest pain (3.1%) and atherosclerosis (0.4%) [15]. However, in our sensitivity analysis, we also did an analysis in which solely myocardial infarction and stroke were included as outcomes to verify the results. The analysis showed that the ORs for the difference between SHIP-TREND und KORA-F4 regions decreased in all models, but remained significant in the age- and sex-adjusted model for the general population. Although the number of coronary angiographies carried out varies widely within Germany, it is comparable between the SHIP-TREND und KORA-F4 regions (SHIP-TREND: 125–152, KORA-F4: 96–124 coronary angiographies per 10,000 persons per year) [15]. Therefore, the result of the sensitivity analysis is very likely attributable to the small number of participants with myocardial infarction or stroke only (SHIP-TREND: 105 and KORA-F4: 109 in the general population, SHIP-TREND: 47 and KORA-F4: 31 in the participants with known diabetes).

## Conclusions

In conclusion, there is an indication that people in the Northeast have a higher prevalence of macrovascular disease than in the South of Germany. In particular, the difference is more relevant in persons with known type 2 diabetes. Prevention strategies and health-care planning need to consider these regional differences in morbidity within Germany for better identification of high-risk groups. Further studies are needed to investigate other risk factors that can explain these regional differences, such as geographical variations in medical care services, regional deprivation or the impact of stress and depression.

## Abbreviations

BMI: Body mass index
CI: Confidence interval
EGFR: Estimated glomerular filtration rate
FRG: Federal Republic of Germany
GDR: German Democratic Republic
HbA1c: Hemoglobin A1c
HDL: High density lipoprotein
HOMA-IR: Homeostatic model assessment of insulin resistance
HsCRP: High-sensitivity C-reactive protein
IQR: Interquartile range
LDL: Low density lipoprotein
NA: Not assessed
OGTT: Oral glucose tolerance test
OR: Odds ratio
SD: Standard deviation
